# Supplementary material for: Resolving the full spectrum of human genome variation using Linked-Reads
Source: Genome Res. 2019 Apr;29(4):635–45. doi: 10.1101/gr.234443.118 (PMC6442396; doi:10.1101/gr.234443.118)
Supplement: Supplemental Material [file supp_gr.234443.118_Supplemental_longranger-2.2.zip › longranger-2.2.tar.bz2!/lr2.2-public/lib/stan/cmdstan/stan/doc/api/html/init__nuts_8hpp_source.html]

Stan: src/stan/common/init\_nuts.hpp Source File


|  |  |
| --- | --- |
|  | Stan  2.5.0  probability, sampling & optimization |


- Main Page
- Related Pages
- Namespaces
- Classes
- Files

- File List
- File Members

All Classes Namespaces Files Functions Variables Typedefs Enumerations Enumerator Friends Macros Pages

- src
- stan
- common

init\_nuts.hpp

Go to the documentation of this file.

1 #ifndef STAN\_\_COMMON\_\_INIT\_NUTS\_HPP

2 #define STAN\_\_COMMON\_\_INIT\_NUTS\_HPP

3

4 #include <stan/mcmc/base\_mcmc.hpp>

5 #include <stan/gm/arguments/argument.hpp>

6 #include <stan/gm/arguments/categorical\_argument.hpp>

7 #include <stan/gm/arguments/singleton\_argument.hpp>

8

9 namespace stan {

10  namespace common {

11

12  template<class Sampler>

13  bool init\_nuts(stan::mcmc::base\_mcmc\* sampler,

14  stan::gm::argument\* algorithm) {

15  stan::gm::categorical\_argument\* hmc

16  = dynamic\_cast<stan::gm::categorical\_argument\*>

17  (algorithm->arg("hmc"));

18

19  stan::gm::categorical\_argument\* base

20  = dynamic\_cast<stan::gm::categorical\_argument\*>

21  (algorithm->arg("hmc")->arg("engine")->arg("nuts"));

22

23  double epsilon

24  = dynamic\_cast<stan::gm::real\_argument\*>(hmc->arg("stepsize"))->value();

25  double epsilon\_jitter

26  = dynamic\_cast<stan::gm::real\_argument\*>(hmc->arg("stepsize\_jitter"))->value();

27  int max\_depth = dynamic\_cast<stan::gm::int\_argument\*>(base->arg("max\_depth"))->value();

28

29  dynamic\_cast<Sampler\*>(sampler)->set\_nominal\_stepsize(epsilon);

30  dynamic\_cast<Sampler\*>(sampler)->set\_stepsize\_jitter(epsilon\_jitter);

31  dynamic\_cast<Sampler\*>(sampler)->set\_max\_depth(max\_depth);

32

33  return true;

34  }

35

36  }

37 }

38

39 #endif

base\_mcmc.hpp

categorical\_argument.hpp

stan::gm::singleton\_argument

**Definition:** singleton\_argument.hpp:44

stan::gm::categorical\_argument::arg

argument \* arg(const std::string name)

**Definition:** categorical\_argument.hpp:143

argument.hpp

stan::gm::categorical\_argument

**Definition:** categorical\_argument.hpp:11

stan::gm::argument::arg

virtual argument \* arg(const std::string name)

**Definition:** argument.hpp:67

singleton\_argument.hpp

stan::gm::argument

**Definition:** argument.hpp:12

stan::common::init\_nuts

bool init\_nuts(stan::mcmc::base\_mcmc \*sampler, stan::gm::argument \*algorithm)

**Definition:** init\_nuts.hpp:13

stan::mcmc::base\_mcmc

**Definition:** base\_mcmc.hpp:13


---

[ Stan Home Page ]

*© 2011–2014,
Stan Development Team.*
